# Evaluation of Not-Activated and Activated PRP in Hair Loss Treatment: Role of Growth Factor and Cytokine Concentrations Obtained by Different Collection Systems

**DOI:** 10.3390/ijms18020408

**Published:** 2017-02-14

**Authors:** Pietro Gentile, John P. Cole, Megan A. Cole, Simone Garcovich, Alessandra Bielli, Maria Giovanna Scioli, Augusto Orlandi, Chiara Insalaco, Valerio Cervelli

**Affiliations:** 1 Plastic and Reconstructive Surgery Department, University of Rome Tor Vergata, Via Courmayeur, No. 102, 00135 Rome, Italy; chiarainsalaco@virgilio.it (C.I.); valeriocervelli@virgilio.it (V.C.); 2 Plastic and Reconstructive Surgery Department, Catholic University, 1005 Tiranna, Albania; 3 Cole Hair Transplant Group, Alpharetta, GA 30004, USA; forhair3@me.com (J.P.C.); megan.cole@colorado.edu (M.A.C.); 4 Institute of Dermatology, Catholic University of the Sacred Heart, 00168 Rome, Italy; simone.garcovich@live.it; 5 Institute of Anatomic Pathology, University of Rome Tor Vergata, 00133 Rome, Italy; alessandrabielli@hotmail.it (A.B.); scioli07@hotmail.it (M.G.S.); orlandi@uniroma2.it (A.O.)

**Keywords:** androgenic alopecia, hair loss, autologous PRP, activated PRP, growth factors

## Abstract

Platelet rich plasma (PRP) was tested as a potential therapy for androgenetic alopecia (AGA) through two different clinical protocols in which one population (18 participants) received half-head treatment with autologous non-activated PRP (A-PRP) produced by CPunT Preparation System (Biomed Device, Modena, Italy) and the other half-head with placebo, and a second separated population in which all participants (*n* = 6, 3 participants per group) received treatment with calcium-activated PRP (AA-PRP) produced from one of two different PRP collection devices (Regen Blood Cell Therapy or Arthrex Angel System). For the A-PRP study, three treatments were administered over 30-day intervals. Trichoscan analysis of patients, three months post-treatment, showed a clinical improvement in the number of hairs in the target area (36 ± 3 hairs) and in total hair density (65 ± 5 hair cm^2^), whereas negligible improvements in hair count (1.1 ± 1.4 hairs) and density (1.9 ± 10.2 hair cm^2^) were seen in the region of the scalp that received placebo. Microscopic evaluation conducted two weeks after treatment showed also an increase in epidermal thickness, Ki67^+^ keratinocytes, and in the number of follicles. The AA-PRP treatment groups received a singular set of injections, and six months after the treatments were administered, notable differences in clinical outcomes were obtained from the two PRP collection devices (+90 ± 6 hair cm^2^ versus −73 ± 30 hair cm^2^ hair densities, Regen versus Arthrex). Growth factor concentrations in AA-PRP prepared from the two collection devices did not differ significantly upon calcium activation.

## 1. Introduction

Androgenetic alopecia (AGA) is a common form of hair loss affecting up to 50% of white men (male-pattern baldness, MPHL) by age 50 and nearly 50% of women (female-pattern hair loss, FPHL) over the course of their lifetime [1,2]. While onset in both males and females may be observed as early as age 18, the progression of hair loss differs markedly between the genders. In men, hair is lost in defined patterns described most commonly by the Norwood and Hamilton scales and often leads to complete baldness [3]; however, FPHL is characterized by diffuse thinning that rarely results in complete baldness [4]. Several therapies have been proposed for the treatment of AGA, but to date, only oral finasteride, topical minoxidil (2% and 5% solutions or foams), and low level laser have been approved by the US Food and Drug Administration (FDA) to combat MPHL [5,6]. Minoxidil 5% foam is also approved by the FDA for female pattern hair loss FPHL. Finasteride, a selective 5-α-reductase inhibitor, has proven largely ineffective in treating FPHL [7], and, given that the drug may cause abnormalities in the external genitalia of male fetuses, is unsuitable for use by pre-menopausal women [8]. Conversely, daily treatment with 1 mg of finasteride has been shown to reduce serum dihydrotestosterone (DHT) levels by 70% and promote the conversion of hair follicles into the anagen (i.e., growth) phase in male AGA patients [9,10], though significant improvements in hair density may require up to one year of treatment and users may experience adverse sexual side effects which may persist after the medication is discontinued [11].

Originally formulated as an antihypertensive, minoxidil is hypothesized to arrest follicular miniaturization and increase anagen duration, both of which counteract the AGA hair loss process [5]. As a result, 60% of users (male or female) show increased hair counts when a 2% topical solution is applied daily [12,13]. Higher concentrations may afford greater increases in non-vellus hair densities, but they are not approved for use in FPHL [4]. Furthermore, they pose an increased risk of skin irritation [14,15] and, like the 2% formulations, must be continued indefinitely to prevent relapse [16]. Although the current pharmacotherapies are largely effective in arresting the progression of AGA, they enable only partial hair regrowth at best and require persistent use to maintain the regenerated hair density; hence, many AGA sufferers seek surgical intervention, which is often supplemented with FDA-approved pharmacological therapies in addition to emerging trends in regenerative medicine. In particular, the contribution of platelets to the inflammatory and healing response has made them an increasingly attractive therapeutic resource in all branches of regenerative medicine owing to the high concentrations of biologically active proteins released from platelet α-granules upon contact with injured tissues [17,18].

Recently, the use of low-level laser therapy (LLLT) has been proposed as a treatment for hair loss and to stimulate hair regrowth in AGA. Eleven studies were evaluated by Afifi et al. [19], which investigated a total of 680 patients, consisting of 444 males and 236 females. Nine out of 11 studies assessing hair count/hair density found statistically significant improvements in both males and females following LLLT treatment. Additionally, hair thickness and tensile strength significantly improved in two out of four studies. Patient satisfaction was reported in five studies.

Pre-operative preparation of platelet concentrate from the whole blood of patients, or autologous platelet rich plasma (A-PRP), is now associated with improved surgical outcomes and lower recurrence rates when incorporated in the treatment protocol for gingival recession and keloid therapies, respectively [20,21]. The range of dermatological applications in which PRP therapies have proven efficacious appears to increase with the delivery of activated autologous PRP (AA-PRP) in place of non-activated A-PRP. When A-PRP is combined with autologous thrombin to yield AA-PRP, for example, clinicians observe healing of chronic wounds and shortened recovery times for deep burns [22,23]. Likewise, laser resurfacing of acne scars affords qualitatively better results with fewer side effects when performed in conjunction with either topical or intradermal application of calcium-activated PRP [24]. Presumably, these clinical improvements may be attributed to the release and concentration of α-granule proteins, including growth factors and cytokines, that promote cellular proliferation and differentiation, angiogenesis, and vascular modeling [25].

In the treatment of AGA, topical application of activated PRP to harvested follicles prior to implantation has already been shown to increase their survival rate by 15% [26]. Moreover, patients treated with calcium gluconate-activated PRP exhibit increased hair density three months post-surgery with terminal hair density (i.e., hair with a diameter >40 μm) increasing by 19% during that time [27]. These findings were confirmed in a study following AGA patients treated with calcium-activated PRP over the course of one year [28]. Three months after the final PRP injection, hair density peaked with a 19% increase over baseline measurements; at the one-year mark, hair density fell to 7% above baseline measurements but this value still constituted a significant increase in hair density compared to the baseline values [28].

Given the positive outlook of AA-PRP [28] as a hair regeneration treatment and the lack of data for the use of native, non-activated A-PRP as an alternative, the primary objective of this work is to determine if similar results in hair growth can be found without activating the PRP prior to delivery. Instead, A-PRP was injected in the absence of a calcium activator, and the release of growth factors and cytokines from α-particles within the platelets was left for the body to stimulate as a natural consequence of the inflammation process in response to the scalp injections. The impact of A-PRP on hair growth was quantified macroscopically (three months post-treatment) by hair count and total hair density, and histologically (two weeks post-treatment) by epidermal thickness and follicle and basal keratinocyte count in A-PRP treatment versus placebo scalp biopsies.

## 2. Results

### 2.1. A-PRP Treatment Population

The hair growth parameters measured 12 weeks after of the first treatment were compared with the baseline measurements made before treatments were administered and between the A-PRP treatment area and the control area, which received placebo injections. Mean total hair counts and hair density measurements for the treatment and control areas are reported in Table 1 as mean ± standard deviation. At the baseline, no statistical differences in hair count or hair density existed between the A-PRP treatment area and control area of the scalp.

**Figure 1:**
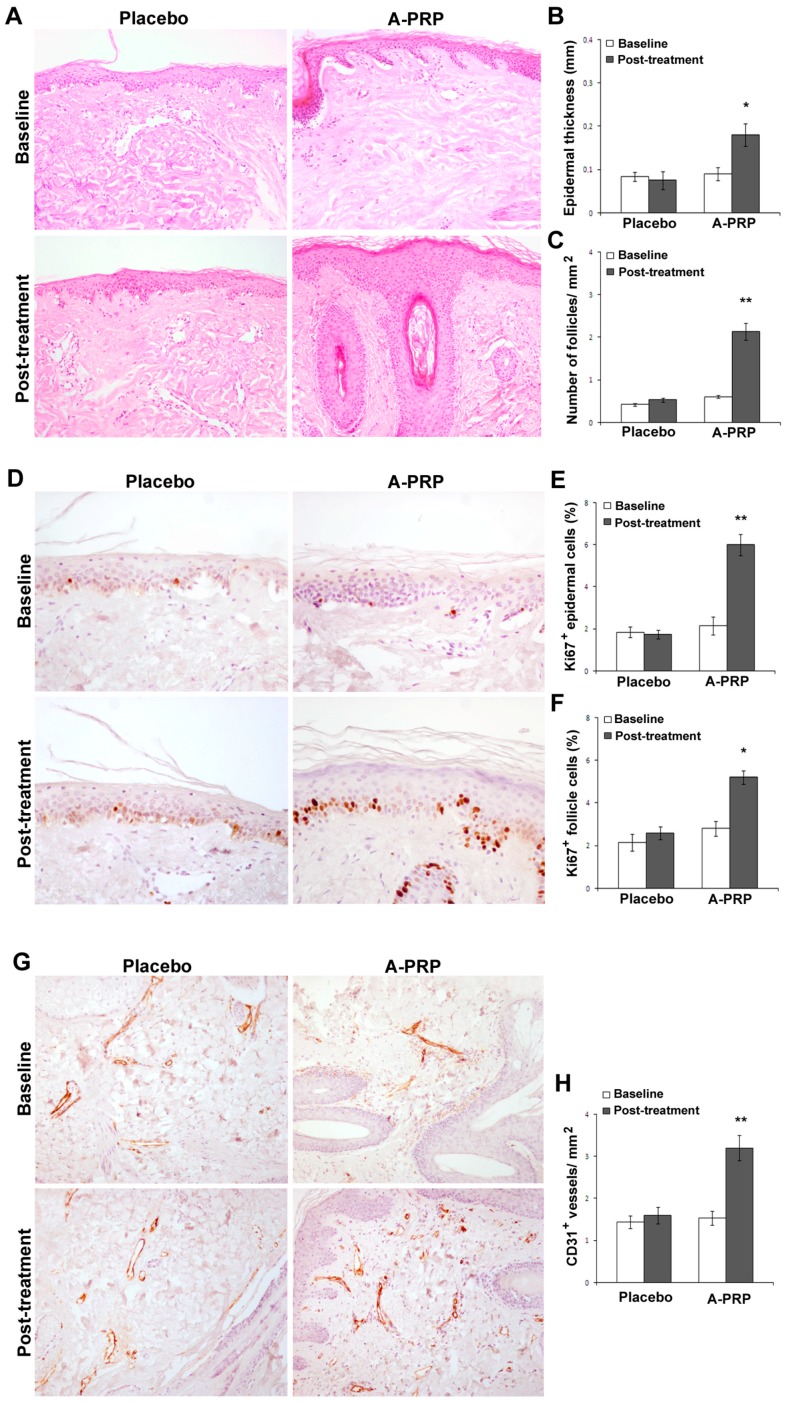
A-PRP treatment increased proliferation of epidermis basal cells and follicular bulge cells as well as vascularization. (**A**) Representative microphotographs of hematoxylin and eosin stained tissue sections from placebo and A-PRP treated scalp biopsies at baseline and two weeks post-treatment (original magnification 100×); (**B**,**C**) Morphometric evaluation of epidermis thickness (mm) and hair follicle density (no follicles mm^−2^); (**D**) Representative microphotographs of Ki67^+^ immunostaining of scalp biopsies from placebo and A-PRP treated patients at baseline and two weeks post-treatment (original magnification 200×); (**E**,**F**) The percentage of proliferating Ki67^+^ epidermal and follicle cells (dark brown nuclei); (**G**) Representative microphotographs of CD31 immunostaining of scalp biopsies from placebo and A-PRP treated patients at baseline and two weeks post-treatment (original magnification 100×); (**H**) Capillary density (CD31^+^ vessels mm^−2^). * and ** indicates *p* < 0.05 and *p* < 0.01, respectively.

On microscopic examination, A-PRP treated scalp demonstrated increased epidermal thickness (Figure 1A,B) accompanied by an increase in the number of follicles (Figure 1A,C), Ki67^+^ basal keratinocytes, and follicular bulge cells relative to baseline levels (Figure 1D–F). Additionally, A-PRP treatment was associated with improved vascularization of hair follicles (Figure 1G,H).

**Figure 2:**
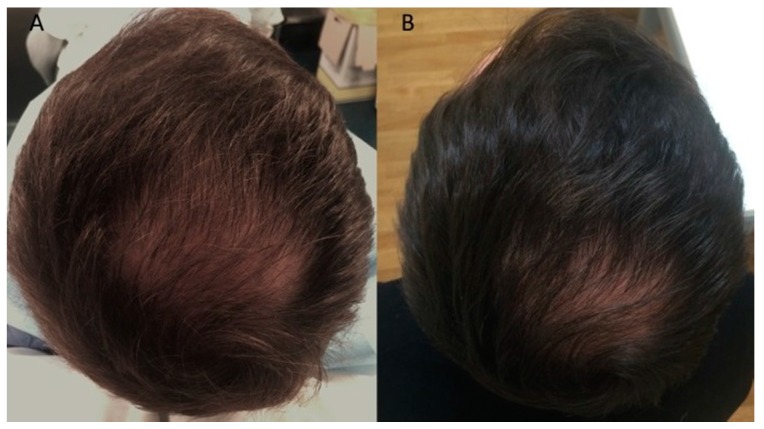
Clinical case of a male patient affected by androgenetic alopecia. (**A**) Pre-operative situation of the frontal, temporal, parietal, and vertex area; (**B**) Post-operative situation after three A-PRP injections with increase in the hair count and hair density.

**Figure 3:**
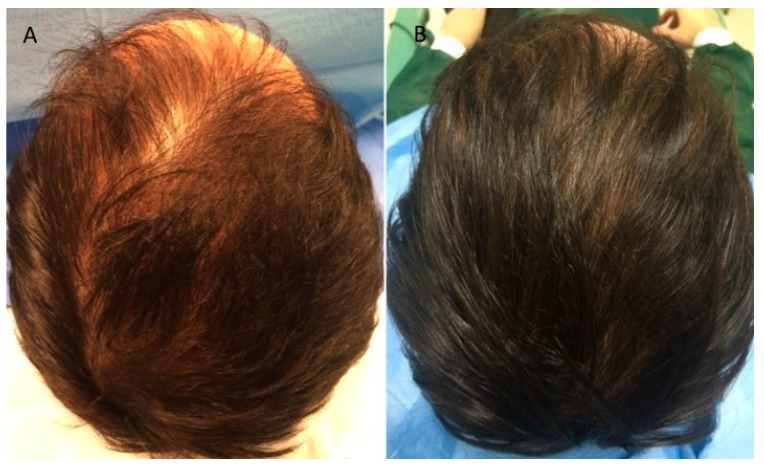
Clinical case of a male patient affected by androgenetic alopecia. (**A**) Pre-operative situation of the frontal, temporal and parietal; (**B**) Post-operative situation after three A-PRP injections with increase in the hair count and hair density.

The results of this study indicate that 12 weeks after treatment with A-PRP mean hair count increases significantly over baseline values. At the 12-week evaluation period, scalp treated with A-PRP displayed an elevated hair count (36 ± 3 hairs) and total hair density (65 ± 5 hair cm^2^), while the region of the scalp that received placebo treatments had negligible changes in hair count (1.1 ± 1 hairs) or hair density (1.9 ± 2 hair cm^2^). These values reflect a 31% ± 2% increase in hair density for the treatment group and less than a 1% increase in hair density for the placebo group. Moreover, both the hair count and hair density parameters represent statistically significant improvements in hair growth for the A-PRP treated scalp (Figure 2A,B and Figure 3A,B) over the placebo treated control group.

Furthermore, at the 12-week evaluation period, scalp treated with three injections of A-PRP displayed a darker coloring of the hair compared to the pre-operative situation. To make a judgment about, the authors used a 1 to 10 scale, where the number 1 was used to indicate black, 5 to indicate a color light brown, and 10 to indicate the lightest blonde (light blond platinum). This assessment made it possible to report a darkening of color, moving from a gradation 4 (brown) (Figure 2A and Figure 3A) to a grade 2 (dark brown) (Figure 2B and Figure 3B).

### 2.2. AA-PRP Treatment Population

Hair growth parameters taken six months after treatment were compared with baseline measurements and between the two test groups, the first having received calcium-activated PRP collected via Regen Cell Therapy and the second with the Arthrex Angel System. The hair density and follicular unit density measurements for both time points and treatments sets are provided in Table 2 as mean ± standard error. At the baseline, no statistical differences in either parameter existed between the two test populations. However, six months after treatment, patients receiving AA-PRP generated from the Arthex Angel System experienced statistically significant increases in both parameters relative to patients who received AA-PRP injections prepared by the Regen Cell Therapy collection system. When the Arthrex Angel system was employed, both hair density and follicular unit density increased, by 56% ± 2% and 30% ± 12%, respectively, while these values decreased from baseline measurements by 23% ± 9% and 2% ± 6%, respectively, when AA-PRP was prepared from Regen Cell Therapy A-PRP.

### 2.3. Growth Factor Quantification

Growth factor concentrations in A-PRP prepared using the CPunT (PDGF-BB, VEGF, and FGF), Regen, and Arthex (PDGF-BB, IGF-1, VEGF, and TGF-β1) collection systems and calcium-activated AA-PRP prepared from the Regen and Arthex collection systems are presented in Table 3 (mean ± standard error). VEGF and IGF-1 levels in Arthrex AA-PRP were higher than those in Regen AA-PRP, whereas PDGF-BB and TGF-β1 levels were higher in Regen AA-PRP than Arthex AA-PRP; however, none of these differences were found to be statistically significant. Comparisons between A-PRP and AA-PRP indicated that higher concentrations of PDGF-BB, TGF-β1, and VEGF are obtained upon activation regardless of the collection system employed, but only the increase in VEGF using the Regen collection system was statistically significant (*p* = 0.0283). Activation had negligible impact on IGF-1 concentration. The CPunT collection system produced A-PRP with higher concentrations of PDGF-BB and VEGF than the Artherx and Regen systems, but these differences were similarly found to be statistically insignificant.

## 3. Discussion

Platelet activation occurs as a natural consequence of hemostasis, and when the process is initiated by thrombin in vivo, platelets recruited to the site of injury release proteins from their α-granules that display a wide range of functional properties from clot induction (adhesive proteins) and platelet aggregation (membrane glycoproteins) to vascularization (cytokines and chemokines) and cell proliferation and differentiation (growth factors) [18]. Moreover, activated platelet-release of the nucleotide adenosine diphosphate (ADP) from dense granules activates additional platelets [29]. Many of the growth factors released from the α-granules of activated platelets have been shown to have a positive impact on hair growth. Specifically, platelet-derived growth factor AA (PDGF-AA) improves the hair inductive activity of dermal papilla cells when applied in combination with fibroblast growth factor 2 (FGF-2) [30]. Insulin-like growth factor-1 (IGF-1) stimulates proliferation of cycling Ki67^+^ basal keratinocytes [31,32], while transforming growth factor β1 (TGF-β1) protects the proliferative potential of basal keratinocytes by inhibiting cell growth and terminal differentiation [33,34]. Vascular endothelial growth factor (VEGF) promotes angiogenesis, and PDGF-BB is a potent chemoattractant for wound macrophages and fibroblasts and stimulates these cells to release endogenous growth factors, including TGF-β1, that promote new collagen synthesis [35].

At the cellular level, dermal papilla cells harvested from human scalp tissue have shown increased proliferation, increased Bcl-2 and FGF-7 levels, activated ERK and Akt proteins, and upregulation of β-catenin when cultured in an activated PRP-supplemented growth medium [36]. Since each of these factors positively influences hair growth through cellular proliferation to prolong the anagen phase (FGF-7) [37], inducing cell growth (ERK activation) [38], stimulating hair follicle development (β-catenin) [39], and suppressing apoptotic cues (Bcl-2 release and Akt activation) [40,41], human scalp injected with PRP should display marked increases in cellular activity. Indeed, histological examination of A-PRP treated scalp from the present study, as well as AA-PRP treated scalp from our previous work [42], provides such clinical evidence. In both patient populations, we observed increases in the number of follicular bulge cells and follicles, epidermal thickening, improved vascularization, and a higher number of Ki67^+^ basal keratinocytes in PRP-treated scalp tissue compared with placebo.

Hair growth at the macroscopic level displayed a similarly positive response to treatment with A-PRP, with participants manifesting significant improvements in hair count and total hair density in the treatment zone over the placebo control zone. Absolute differences between 12-week follow-up counts and baseline counts for these hair growth parameters were higher in the A-PRP treatment population in this study than in the AA-PRP treatment population in our previous trial [42]. In particular, 12-week hair density measurements for patients treated with A-PRP and AA-PRP were 65 ± 5 and 28 ± 4 hair cm^2^, respectively. These values constitute a 31% ± 2% increase in hair density when A-PRP treatment is applied versus a 19% ± 3% increase in hair density when AA-PRP treatment is applied, a statistically significant difference in hair growth (*p* = 0.0029). The larger improvement in hair growth parameters for A-PRP over AA-PRP may reflect the greater efficiency of in vivo thrombin to activate platelets and the body to distribute the contents of activated platelets compared with in vitro calcium activation and injection. Moreover, delivery of A-PRP may enable production of thromboxane A_2_ (TXA2) by the platelets once they are activated in vivo, which would activate additional platelets and amplify platelet aggregation [43]. Since platelets are inherently fragile, the added processing associated with calcium-activation might damage the cells to a degree that they are no longer able to synthesize TXA2.

In an effort to compare the relative value of PRP collected from systems available to clinicians, two additional treatment populations were incorporated in this study. In the first, AA-PRP was prepared using A-PRP collected by an Arthex Angel System, and in the second, AA-PRP was prepared from A-PRP derived from a Regen Cell Therapy device. Hair density increased by 90 ± 6 hair cm^2^ over baseline measurements six months after treatment with Arthex-derived AA-PRP, the largest improvement in hair density observed in this study (56% ± 2% increase in hair density), but fell by 73 ± 30 hair cm^2^ in participants who received AA-PRP collected with the Regen device. The 56% improvement in hair density from treatment with Arthrex AA-PRP is a statistically significant improvement in hair growth relative to the 19% and 31% increases in hair density when scalp is treated with CPunT AA-PRP (*p* < 0.0001) or A-PRP (*p* = 0.0005), respectively.

The disparity in treatment outcomes observed in this study may likely be attributed to the differences in the concentration and quality of platelets by the various collection systems in addition to the relative abundance of each growth factor within the platelet α-granules. ELISA studies performed herein provide a limited concentration profile of growth factors with documented influence on hair growth (i.e., PDGF-BB, TGF-β1, IGF-1, and VEGF; see Table 3) available in A-PRP and AA-PRP lysate when the Regen and Arthrex collection systems are used and in A-PRP when the CPunT collection system is used. A-PRP growth factor concentrations are nearly identical for all three systems, but minor differences are detected upon calcium activation. The Regen system is higher in PDGF-BB and TGF-β, but the Arthrex system is higher in IGF-1 and VEGF. Therefore, patients treated with Regen-derived AA-PRP should exhibit faster rates of collagen deposition and reduced numbers of Ki67^+^ basal keratinocytes in their epidermis, while patients treated with Arthrex-derived AA-PRP should have increased numbers of Ki67^+^ basal keratinocytes and improved vascularization upon histological examination. Given the expected outcomes in scalp epidermis, changes in the macroscopic hair growth parameters are more likely for the Arthrex-treatment group, and indeed, this is the treatment group that responded best to AA-PRP injections.

VEGF levels for the C-PunT-derived A-PRP fell in between those of A-PRP and AA-PRP for the Arthrex and Regen collection systems. Therefore, one might anticipate scalp biopsies from patients treated with the C-PunT A-PRP to display an intermediate increase in vascularization as well as improvements in hair density below that of Arthrex AA-PRP treated scalp. However, the improvements in hair density may require a combined high concentration of VEGF and IGF-1 given that six-month hair density measurements dropped in patients treated with Regen AA-PRP but increased after 12 weeks for CPunT A-PRP treated patients and the concentration of VEGF in Regen AA-PRP exceeded that in CPunT A-PRP. Unfortunately, scalp histopathological examination of AA-PRP treated scalp or measurement of IGF-1 levels in C-PunT A-PRP were not performed to confirm this assumption.

## 4. Materials and Methods

### 4.1. Study Overview

Two different clinical studies (A-PRP and AA-PRP) were conducted by plastic surgeons, biologists, and pathologists of the University of Rome Tor Vergata, a dermatologist of the Catholic University of the Sacred Heart of Rome, and Cole Hair Transplant Group (Atlanta, GA, USA). The primary outcomes for the placebo-controlled, half-head group study with A-PRP treatment were hair count and hair density based on computerized trichogram. The secondary outcomes were microscopic evaluation of the epidermis thickness, change in follicle quantity, and qualitative evaluation of safety and feasibility in PRP-treated skin biopsies. Evaluators of computerized trichograms were blinded to the treatment methods. The outcomes for patients treated with AA-PRP were hair density and follicular unit density.

MPHL diagnoses were established on the basis of a detailed medical history (i.e., screening for drugs linked to hair loss), clinical examination, and trichoscopic features (i.e., >20% variability in hair diameter between affected and unaffected areas). Patients were clinically diagnosed with MPHL upon presentation of an increase in miniaturized terminal hair and/or a reduced number of hairs on physical examination and phototrichograms, along with negative hair pull tests. Laboratory tests were performed to exclude alternative causes of hair loss, such as poor nutrition, anemia (i.e., complete blood count, serum iron, serum ferritin, total iron binding capacity, and folic acid), thyroid dysfunction (i.e., triiodothyronine (T_3_), free T_3_ (FT_3_), thyroxine (T_4_), free T_4_ (FT_4_), and thyroid-stimulating hormone (TSH), antithyroid peroxidase, and testosterone), and syphilis (i.e., a venereal disease research laboratory blood test). Urinalysis was used to detect levels of 17-idrocorticosteroid, 17-ketosteroid, dehydroepiandrosterone (DHEA), free cortisol, pregnanetriol (PTL), and testosterone (T) in all participants. Finally, circulating levels of cortisol, dihydrotestosterone (DHT), DHEA, Δ^4^-androstenedione, 17-hydroxyprogesterone, 3-α-diol glucuronide, prolactin, and gonadotropins (i.e., FSH and LH) were measured on all participants. The stage of individual participant alopecia was evaluated according to the Hamilton–Norwood scale. The study protocol complied with the Declaration of Helsinki, and all patients provided written informed consent before participating in the study.

### 4.2. A-PRP Patient Population and Randomization

This study enrolled 18 male patients aged 19–63 years who displayed MPHL in stage 2–4 as determined by the Norwood–Hamilton classification scale (Table 4); those with advanced hair loss in stage 5–7 were excluded. Additional exclusion factors were set based on systemic and local criteria. Specifically, systemic criteria for exclusion included evidence of platelet disorders, thrombocytopenia, bone marrow aplasia, prior antiaggregation therapy, uncompensated diabetes, sepsis, immunosuppression, and cancer, as well as use of pharmacological therapeutics targeting MPHL (i.e., finasteride, dutasteride, or antiandrogens) in the previous 12 months. Localized exclusion criteria included use of topical treatments for MPHL (i.e., minoxidil, prostaglandin analogs, retinoids, or corticosteroids) in the previous 12 months and withdrawal of informed consent. Three patients with a propensity for keloids were also excluded. All of the participants included in this study were assessed by two experts in plastic surgery and deemed suitable for PRP injections.

The treatment allocation sequence was generated using an online randomization generator and was concealed by an individual unrelated to the trial management group. The participants, study personnel, and outcome assessors were all blinded to treatment allocation, and blinding was maintained until all data had been analyzed.

### 4.3. AA-PRP Patient Population

This study enrolled six male patients aged 35–58 years who displayed MPHL in stage 3A–3V as determined by the Norwood–Hamilton classification scale (Table 5). The patients were then divided into two groups; the first was treated with AA-PRP produced using a Regen Blood Cell Therapy tubes while the second was treated with AA-PRP prepared with the Arthrex Angel System. A second group of five patients (three male and two female aged 20–60 years) with no apparent hair loss was selected solely for analyzing growth factor concentrations in AA-PRP using the two collection procedures listed above. Written informed consent was obtained from all participants.

### 4.4. A-PRP Procedures

#### 4.4.1. A-PRP Preparation and Delivery

**Figure 4:**
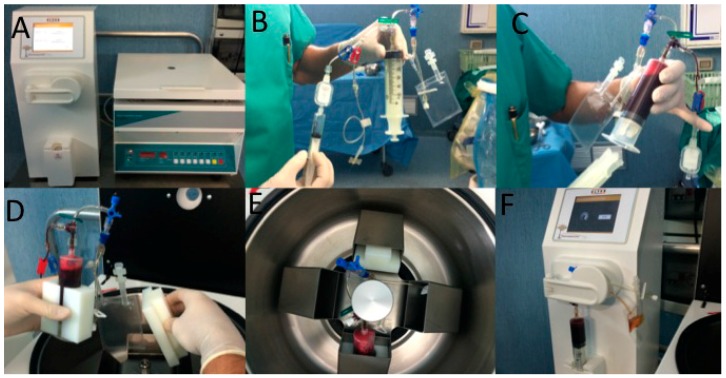
A-PRP preparation by C-PunT System (Biomed Device, Modena, Italy). (**A**) Centrifuge and light selector device; (**B**) C-PunT kit; (**C**) Whole blood (55 mL) was collected from a peripheral vein using sodium citrate as an anticoagulant; (**D**) Whole blood contained in a syringe was inserted in a centrifuge; (**E**) Centrifugation (1200 rpm for 10 min); (**F**) The autologous platelet suspension (Platelet Poor Plasma—PPP and Platelet Rich Plasma—PRP)-obtained (23 mL) was inserted in a light selector device and at the end of the procedure, 9 mL of A-PRP was harvested.

Whole blood (55 mL) was collected from a peripheral vein using sodium citrate as an anticoagulant (Figure 1B,C). A-PRP (23 mL) was produced for all cases using the CPunT Preparation System (Biomed Device, Modena, Italy) under the approval of the transfusional service. Following centrifugation (1200 rpm for 10 min) (Figure 4A,D,E), the A-PRP was inserted in a light selector device (Figure 4F), and at the end of the procedure, 9 mL of A-PRP was harvested. Microscopic platelet counts were performed on the A-PRP collected from all participants.

**Figure 5:**
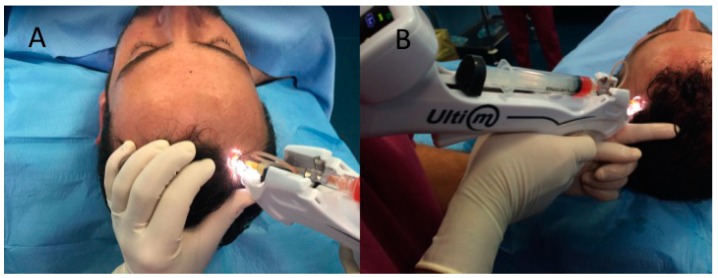
Interfollicular A-PRP injections by Ultim gun (Anti-Aging Medical Systems). (**A**) Interfollicular A-PRP injections (0.2 mL·cm^−2^) were administered to select areas of the scalp at a depth of 5 mm; (**B**) Ultim gun equipped with a 30-gauge, 10 mL Luer lock syringe in three sessions spaced 30 days apart.

For each patient, the scalp affected by hair loss was divided into four areas (frontal, parietal, vertex, and occipital) and cleansed with 70% alcohol; local anesthesia was not injected in the treated areas. Interfollicular A-PRP injections (0.2 mL·cm^−2^) were administered to select areas of the scalp at a depth of 5 mm using an Ultim gun (Anti-Aging Medical Systems, Montrodat, France) equipped with a 30-gauge, 10 mL Luer lock syringe (Figure 5A,B) in three sessions spaced 30 days apart. In patients with hair loss localized to the frontal and parietal regions, A-PRP injections were delivered exclusively to the frontal scalp while placebo injections (i.e., physiological saline) were injected in the parietal regions. Likewise, for patients with hair loss limited to the parietal and vertex regions, A-PRP was injected in the parietal region, and placebo was injected in the vertex region of the scalp. Equivalent numbers of A-PRP and placebo injections were made.

#### 4.4.2. Assessment of Hair Growth Parameters

Photographs of the areas of a sample scalp treated with A-PRP are shown in Figure 5. The effects of A-PRP and placebo treatments on hair growth were assessed in all patients with the help of global photography, physician’s and patient’s global assessment scale, and standardized phototrichograms, which were conducted on all scalps by a trained evaluator using video-epiluminescence microscopy (FotoFinder Trichovision, FotoFinder Systems, Inc., Columbia, CA, USA) in conjunction with digital image analysis (TrichoScan, Tricolog GmbH, Freiburg, Germany). TrichoScan is a digital software-supported epiluminescence technique for measuring hair count (number of hairs per 0.65 cm^2^), hair density (number of hairs per cm^2^), hair diameter, anagen-to-telogen ratio, and vellus hair-to-terminal hair ratio. To determine the quality of hair leading to an increased hair density, it was important to differentiate the number of terminal and vellus hairs.

In the hair count, performed by TrichoScan analysis, all hairs with a diameter >40 μm were included and categorized as terminal hairs; those with lesser diameter categorized as vellus hairs were not included. In all patients, two translational areas of hair loss, one at the border of the A-PRP treatment half and a second along the border of the placebo half, were demarcated with a semi-permanent tattoo for hair counting and follow-up trichogram analysis. In the target area, hairs were clipped and dyed brown for 10 min to improve the hair contrast for the analytic software. The evaluator of the computerized trichogram analysis was blinded with respect to the treatment and placebo areas of the scalp and was not involved in administering the interfollicular injections. All patients were subjected to these evaluation methods upon their initial visit and at a follow-up visit 12 weeks after the final injections were delivered.

#### 4.4.3. Histological Evaluation

Incisional punch biopsies (diameter: 3 mm) of the hair skin were obtained at baseline and after two weeks from the last PRP treatment, and fixed in buffered formalin. Morphometric analysis was performed on hematoxylin-and eosin-stained paraffin serial 5 μm-sections. In particular, the thickness of the epidermis was calculated on five random chosen fields in the histological preparation at magnification 400× and analyzed using Scion Image software (Scion Corporation, Frederick, MD, USA, available on: http://www.scioncorp.com). The mean value of the five measurements was calculated for each subject. The number of follicles per mm^2^ was calculated according to the unbiased counting method [44]. Briefly, consecutive paired sections (90 consecutive sections per biopsy) were analyzed, and follicles that were observed in the first section of the pair but not in the subsequent section were counted. Thus, the hair follicle counts represent the actual number within the portion of the biopsy generated by the 90 sections viewed. All samples were cut longitudinally at the skin surface and embedded paying attention to the correct orientation.

#### 4.4.4. Immunohistochemistry

Immunohistochemistry was performed using mouse monoclonal anti-Ki67 and anti-CD31 (Dako, Agilent Technologies, Glostrup, Denmark; available on: http://www.dako.com) [45,46,47,48]. The percentage of Ki67^+^ cells in the basal layer of the epidermis and in the outer root sheath of hair follicles, and the number of vessels per mm^2^ were calculated according to morphometric criteria.

#### 4.4.5. Growth Factors Quantification

Levels of PDGF-BB, VEGF and FGF were quantified using Bio-Plex system (Bio-Rad Laboratories, Hercules, CA, USA) (Table 6). At least two independent repetitions in duplicate were made per sample. Concentrations of the analytes were expressed in pg/mL. A standard curve ranging on average from 0.15 to 3700 pg/mL (High Photomultiplier Tube Setting—PMT setting) was prepared and then fitted by Bio-Plex Manager software (version 6.1, Bio-Rad Laboratories, Hercules, CA, USA).

#### 4.4.6. Statistical Analysis

Statistical analyses of the data were performed using the Statistical Package for the Social Sciences (SPSS), version 19.0 (IBM, New York, NY, USA; available on: http://www-01.ibm.com). The normality of quantitative variables was tested by the Kolmogorov–Smirnov test. Hair growth parameters are expressed as mean ± standard error. All tests were 2-tailed and statistical significance was considered for *p* < 0.05.

### 4.5. AA-PRP Procedures

#### 4.5.1. AA-PRP Preparation and Delivery

Regen Blood Cell Therapy (BCT) tubes were used to prepare A-PRP (15 mL, 5 mL per BCT tube) from whole blood (24 mL) taken from a peripheral vein using sodium citrate as an anticoagulant. The top 2 mL of A-PRP from each tube was then discarded, giving 9 mL of A-PRP with a five-fold increase in platelet concentration over whole blood. Similarly, the Arthrex Angel system was used to prepare A-PRP (3 mL) from 120 mL of whole blood when the instrument hematocrit level was set to 2%. The A-PRP was then combined with 5 mL of platelet poor plasma to produce 8 mL of A-PRP with a five-fold increase in platelet concentration over whole blood. A-PRP collected from both systems was then activated through the addition of 10% (*v*/*v*) calcium gluconate, which was immediately injected into the treatment zone through a 1 mL Luer lock syringe equipped with a 25-gauge needle. Multiple 4 cm^2^ sections (2 cm × 2 cm squares) of frontal scalp constituted the treatment zone, and each received 1 mL of AA-PRP.

#### 4.5.2. Assessment of Hair Growth Parameters

The boundaries of the 4 cm^2^ AA-PRP treatment area were demarcated with a tattoo, and the hair within these boundaries was trimmed and dyed for enhanced contrast. Images of the test region were then captured using a dermlite pro attached to a Sony DSC-560 camera and a 10 mm^2^ reticule at their initial visit and at a follow-up visit six months after the treatment was administered. Hair density and follicular unit density measurements were made from enlarged images of the baseline and AA-PRP treated scalp.

#### 4.5.3. Growth Factor Quantification

Regen Blood Cell Therapy tubes were used to prepare PRP samples from all five subjects, and the Arthrex Angel System was used to prepare PRP samples from one male and one female subject. Once collected, the PRP was either left untreated or activated using a conventional calcium gluconate-based method in which 1 mL of PRP from each test sample was incubated with 10% (*v*/*v*) calcium gluconate at room temperature for 10 min or until a firm blood clot had formed. The untreated PRP and activated PRP samples were then centrifuged for 10 min at 1967× *g*, and the supernatant was recovered and stored at 4 °C prior to testing.

Levels of PDGF-BB, IGF-1, TGF-β1, and VEGF were quantified using commercially-sourced enzyme-linked immunosorbent assay (ELISA) kits (Table 6). Briefly, standards and samples were added to a 96-well microplate pre-coated with an antibody against the target growth factor. Growth factors present within the standard or test samples were bound, and unbound substances were rinsed away. An enzyme-linked polyclonal antibody, specific for the target growth factor, was added in excess and the unbound antibody was rinsed away. A substrate solution was then added and color developed in proportion to the quantity of bound growth factor. After the color development was stopped, the absorbance was measured at 450 nm using a μQuant microplate reader (Bio-Tek, Winooski, VT, USA). Growth factor concentrations were determined from standard curves with the aid of GraphPad Prism curve-fitting software. For IGF-1 and TGF-β1, the data was linearized by plotting the log(concentration) versus the log(OD450), and a best fit was found via linear regression analysis. For all others, a nonlinear four-parameter logistic curve fit was performed. Growth factor measurements for the Arthrex Angel sytem contained two subjects (*n* = 2); for Regen-derived growth factor measurements, *n* = 5.

#### 4.5.4. Statistical Analysis

Statistically significant differences in hair growth parameters between Regen- and Arthrex-derived AA-PRP treatments were determined by unpaired *t*-tests (α = 0.05). Platelet growth factor and cytokine concentrations are expressed as mean ± standard error (*n* = 3). Unpaired *s*-tests were also used to determine statistical significance between data sets (α = 0.05).

## 5. Conclusions

From these experimental results, we demonstrated that A-PRP collected with a CPunT Preparation System and AA-PRP prepared with the aid of an Arthrex Angel system represent viable AGA treatment options. In fact, patients treated with A-PRP were found to have greater increases in hair count and total hair density than patients treated with AA-PRP using an identical PRP collection device, indicating that PRP does not need to be activated when a CPunT Preparation System is employed. Evaluation of growth factor concentrations within AA-PRP collected using the Regen and Arthrex systems did not display statistically significant differences, although the absolute quantities did vary, and the clinical results significantly favored the Arthex device. Therefore, the authors recommend that future clinical trials incorporate a more thorough evaluation of growth factor concentrations in their PRP.

## Figures and Tables

**Table 1:** Relevant hair-growth parameters assessed by Trichoscan analysis for the A-PRP treatment and placebo negative control half-head areas at baseline and after 12 weeks.

Hair Growth Parameter	Time	A-PRP Treatment Area	Control Area
Hair Count	Baseline	122 ± 10	126 ± 9
	12 weeks	158 ± 11	127 ± 9
Hair density, No. per cm^2^	Baseline	218 ± 17	225 ± 15
	12 weeks	282 ± 20	227 ± 16

Data are presented as mean ± standard error.

**Table 2:** Hair-growth parameters assessed by photography for the AA-PRP treatment groups at baseline and after six months.

Hair Growth Parameter	Time	Regen AA-PRP	Arthrex AA-PRP
Hair Density, no. per cm^2^	Baseline	283 ± 64	160 ± 6
	6 months	210 ± 40	250 ± 12
Follicular Unit Density, No. per cm^2^	Baseline	103 ± 19	77 ± 12
	6 months	103 ± 24	97 ± 7

Data are presented as mean ± standard error.

**Table 3:** Growth factor and cytokine concentrations from A-PRP obtained from Regen, Arthrex, and C-PunT collection systems and for AA-PRP obtained from Regen and Arthrex collection systems.

Protein	Collection System	A-PRP	Ca^2+^ AA-PRP
PDGF-BB (ng·mL^−1^)	Regen	1.2 ± 0.3	4.0 ± 2
	Arthrex	1.1 ± 0.6	3.0 ± 1
	C-PunT	1.8 ± 0.4	-
TGF-β1 (ng·mL^−1^)	Regen	11 ± 2	15 ± 3
	Arthrex	12 ± 1	13 ± 0
IGF-1 (ng·mL^−1^)	Regen	130 ± 20	140 ± 20
	Arthrex	150 ± 40	150 ± 60
VEGF (pg·mL^−1^)	Regen	61 ± 20	210 ± 40
	Arthrex	61 ± 20	260 ± 70
	C-PunT	100 ± 20	-
FGF (pg·mL^−1^)	C-PunT	280 ± 60	-

Data are represented as mean ± standard error.

**Table 4:** Summary of A-PRP treated patient population.

Case No.	Age, Years	Hamilton–Norwood Classification Stage	Injection Site
1	29	2A	Frontal + Temporal
2	34	2A	Frontal + Temporal
3	40	3A	Frontal + Temporal
4	42	3V	Frontal + Vertex
5	31	3A	Frontal + Temporal
6	39	3V	Frontal + Vertex
7	47	3V	Frontal + Vertex
8	40	3A	Frontal + Temporal
9	36	2A	Frontal + Temporal
10	51	4	Frontal + Temporal + Vertex + Parietal
11	35	3A	Frontal + Temporal
12	31	3A	Frontal + Temporal
13	43	3V	Frontal + Vertex
14	36	3A	Frontal + Temporal
15	32	3	Frontal + Temporal
16	61	4A	Frontal + Temporal + Vertex + Parietal
17	27	2A	Frontal + Temporal
18	20	2	Frontal

**Table 5:** Summary of AA-PRP treated patient population.

Case No.	Age, Years	Hamilton-Norwood Classification Stage	Test Group
1	43	3V	Regen
2	45	3V	Regen
3	40	3V	Regen
4	58	3V	Arthrex
5	35	3A	Arthrex
6	24	3A–3V	Arthrex

**Table 6:** Commercially-sourced ELISA kits used to quantify platelet growth factors.

Target Protein	ELISA Assay Kit Specifications
PDGF-BB	cat # EHPDGFB, Thermo Scientific
VEGF	cat # KGH011, Novex Life Technologies
IGF-1	cat # DG100, R&D Systems
TGF-β1	cat # KAC1688, Invitrogen Life Technologies
